# NASICON-type Na_3_V_2_(PO_4_)_3_

**DOI:** 10.1107/S1600536810002801

**Published:** 2010-01-27

**Authors:** Igor V. Zatovsky

**Affiliations:** a Department of Inorganic Chemistry, Taras Shevchenko National University, 64 Volodymyrska str., 01601 Kyiv, Ukraine

## Abstract

Single crystals of the title compound, tris­odium divanadium(III) tris­(orthophosphate), were grown from a self-flux in the system Na_4_P_2_O_7_–NaVP_2_O_7_. Na_3_V_2_(PO_4_)_3_ belongs to the family of NASICON-related structures and is built up from isolated [VO_6_] octa­hedra (3. symmetry) and [PO_4_] tetra­hedra (.2 symmetry) inter­linked *via* corners to establish the framework anion [V_2_(PO_4_)_3_]^3−^. The two independent Na^+^ cations are partially occupied [site-occupancy factors = 0.805 (18) and 0.731 (7)] and are located in channels with two different oxygen environments, *viz* sixfold coordination for the first (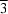. symmetry) and eightfold for the second (.2 symmetry) Na^+^ cation.

## Related literature

For structures and properties of complex phosphates with general formula Na_3_ *M* ^III^_2_(PO_4_)_3_ (*M* ^III^ = Sc, Fe, Cr), see: Collin *et al.* (1986 ▶); Genkina *et al.* (1991 ▶); Lazoryak *et al.* (1980 ▶); Lucazeau *et al.* (1986 ▶); Masquelier *et al.* (1992 ▶); Susman *et al.* (1983 ▶). For preparation of NaVP_2_O_7_ which was used as an educt for crystal growth of the title compound, see: Zatovsky *et al.* (1999 ▶).

## Experimental

### 

#### Crystal data

Na_3_V_2_(PO_4_)_3_
*M* *_r_* = 455.76
Trigonal, 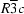

*a* = 8.7288 (2) Å
*c* = 21.8042 (7) Å
*V* = 1438.73 (7) Å^3^
*Z* = 6
Mo *K*α radiation
μ = 2.66 mm^−1^
*T* = 293 K
0.20 × 0.15 × 0.10 mm

#### Data collection

Oxford Diffraction Xcalibur-3 CCD diffractometer
Absorption correction: multi-scan (Blessing, 1995 ▶) *T*_min_ = 0.635, *T*_max_ = 0.780
12580 measured reflections
1331 independent reflections
1153 reflections with *I* > 2σ(*I*)
*R*_int_ = 0.063

#### Refinement

*R*[*F* ^2^ > 2σ(*F* ^2^)] = 0.032
*wR*(*F* ^2^) = 0.075
*S* = 1.1
1331 reflections
37 parameters
Δρ_max_ = 1.12 e Å^−3^
Δρ_min_ = −0.74 e Å^−3^

### 

Data collection: *CrysAlis CCD* (Oxford Diffraction, 2006 ▶); cell refinement: *CrysAlis CCD*; data reduction: *CrysAlis RED* (Oxford Diffraction, 2006 ▶); program(s) used to solve structure: *SHELXS97* (Sheldrick, 2008 ▶); program(s) used to refine structure: *SHELXL97* (Sheldrick, 2008 ▶); molecular graphics: *DIAMOND* (Brandenburg, 1999 ▶); software used to prepare material for publication: *WinGX* (Farrugia, 1999 ▶) and *enCIFer* (Allen *et al.*, 2004 ▶).

## Supplementary Material

Crystal structure: contains datablocks global, I. DOI: 10.1107/S1600536810002801/wm2293sup1.cif

Structure factors: contains datablocks I. DOI: 10.1107/S1600536810002801/wm2293Isup2.hkl

Additional supplementary materials:  crystallographic information; 3D view; checkCIF report

## Figures and Tables

**Table 1:** Selected bond lengths (Å)

V1—O2^i^	1.9693 (10)
V1—O1^ii^	2.0271 (9)
Na1—O1	2.5045 (11)
Na2—O1^ii^	2.3883 (12)
Na2—O1^i^	2.4448 (19)
Na2—O2^iii^	2.6280 (16)
Na2—O2^iv^	2.8352 (19)
P1—O2^v^	1.5227 (12)
P1—O1	1.5358 (10)

Symmetry codes: (i) 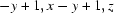; (ii) 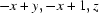; (iii) 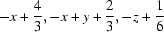; (iv) 
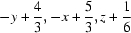
; (v) 
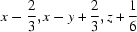
.

**Fig. 1.**
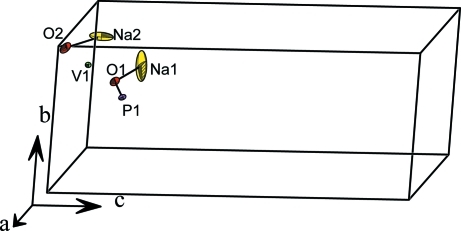
The asymmetric unit of Na3V2(PO4)3 with displacement ellipsoids at the 50% probability level.

**Fig. 2.**
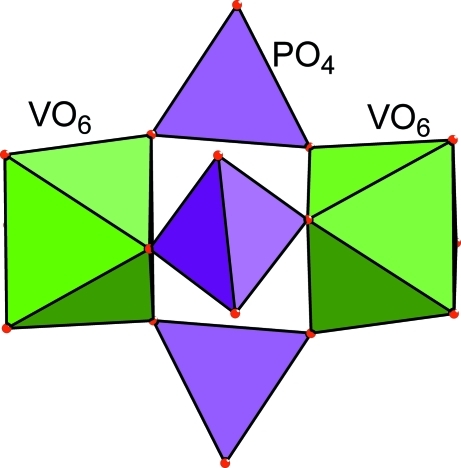
The central building block [V2(PO4)3] in the structure of compound (I).

**Fig. 3.**
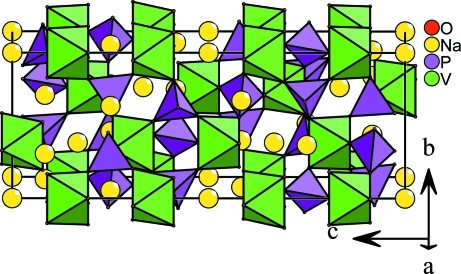
The view of the structure of compound (I) on the ab plane.

## supplementary crystallographic information

### Comment

The structures and properties of complex phosphates with general formula Na_3_*M*^III^_2_(PO_4_)_3_ (*M*^III^ - Sc, Fe, Cr) have been intensively investigated, for instance: (Collin *et al.*, 1986; Genkina *et al.*, 1991; Lazoryak *et al.*, 1980; Lucazeau *et al.*, 1986; Masquelier *et al.*, 1992; Susman *et al.*, 1983). The structure of NASICON-type Na_3_V_2_(PO_4_)_3_, (I),  is reported here.

In the asymmetric unit of (I) (Fig. 1) there is one V and one P atom, while other,atoms (Na and O) are represented in two distinct positions each. The main building block of (I) (Fig. 2) involves two VO_6_ octahedra interlinked by three phosphate groups lying along the *c* axis. As a result of the block aggregation, a three-dimensional framework with an overall composition of [V_2_(PO_4_)_3_]^3-^ is organized (Fig. 3).

Sodium atoms are located in the voids of the framework with six- (position 6*b*) and eightfold (18*b*) coordination when a cut-off distance of 2.9 Å is considered.

### Experimental

Crystals of (I) were obtained in the system Na_4_P_2_O_7_—NaVP_2_O_7_ using a high-temperature crystallization technique. Initial NaVP_2_O_7_ was prepared in accordance to Zatovsky *et al.* (1999). A thoroughly ground mixture of Na_4_P_2_O_7_ and NaVP_2_O_7_ (ratio 1:3) was heated up to 1343 K and then kept for 2 h in a sealed silica tube under vacuum. Then it was cooled down to 823 K with a rate of 5 K/h and left in the furnace to reach room temperature. The final product, green prismatic crystals, was leached out from the solidified melt with boiling water.

### Refinement

After refinement of a basic model and an accurate definition of the atom positions, the occupancy of Na1 and Na2 sites were refined freely and were finally restrained to meet the criterion for charge balance. The highest peak and the deepest hole in the final difference map is located at 0.00 Å from Na1 (1.12 e/Å^3^) and 0.60 from Na2 (-0.74 e/Å^3^) respectively.

### Crystal data

**Table:**

Na_3_V_2_(PO_4_)_3_	*D*_x_ = 3.156 Mg m^−^^3^
*M**_r_* = 455.76	Mo *K*α radiation, λ = 0.71073 Å
Trigonal, *R*3*c*	Cell parameters from 12580 reflections
Hall symbol: -R 3 2"c	θ = 3.3–45.0°
*a* = 8.7288 (2) Å	µ = 2.66 mm^−^^1^
*c* = 21.8042 (7) Å	*T* = 293 K
*V* = 1438.73 (7) Å^3^	Prism, green
*Z* = 6	0.20 × 0.15 × 0.10 mm
*F*(000) = 1320	

### Data collection

**Table:**

Oxford Diffraction Xcalibur-3 CCD diffractometer	1331 independent reflections
Radiation source: fine-focus sealed tube	1153 reflections with *I* > 2σ(*I*)
graphite	*R*_int_ = 0.063
φ and ω scans	θ_max_ = 45°, θ_min_ = 3.3°
Absorption correction: multi-scan (Blessing, 1995)	*h* = −17→16
*T*_min_ = 0.635, *T*_max_ = 0.780	*k* = −17→17
12580 measured reflections	*l* = −43→41

### Refinement

**Table:**

Refinement on *F*^2^	Primary atom site location: structure-invariant direct methods
Least-squares matrix: full	Secondary atom site location: difference Fourier map
*R*[*F*^2^ > 2σ(*F*^2^)] = 0.032	*w* = 1/[σ^2^(*F*_o_^2^) + (0.0264*P*)^2^ + 5.1429*P*] where *P* = (*F*_o_^2^ + 2*F*_c_^2^)/3
*wR*(*F*^2^) = 0.075	(Δ/σ)_max_ < 0.001
*S* = 1.1	Δρ_max_ = 1.12 e Å^−^^3^
1331 reflections	Δρ_min_ = −0.73 e Å^−^^3^
37 parameters	Extinction correction: *SHELXL97* (Sheldrick, 2008)
0 restraints	Extinction coefficient: 0.0056 (4)

### Special details

**Table:**

Geometry. All e.s.d.'s (except the e.s.d. in the dihedral angle between two l.s. planes) are estimated using the full covariance matrix. The cell e.s.d.'s are taken into account individually in the estimation of e.s.d.'s in distances, angles and torsion angles; correlations between e.s.d.'s in cell parameters are only used when they are defined by crystal symmetry. An approximate (isotropic) treatment of cell e.s.d.'s is used for estimating e.s.d.'s involving l.s. planes.
Refinement. Refinement of *F*^2^ against ALL reflections. The weighted *R*-factor *wR* and goodness of fit *S* are based on *F*^2^, conventional *R*-factors *R* are based on *F*, with *F* set to zero for negative *F*^2^. The threshold expression of *F*^2^ > σ(*F*^2^) is used only for calculating *R*-factors(gt) *etc*. and is not relevant to the choice of reflections for refinement. *R*-factors based on *F*^2^ are statistically about twice as large as those based on *F*, and *R*- factors based on ALL data will be even larger.

### Fractional atomic coordinates and isotropic or equivalent isotropic displacement parameters (Å^2^)

**Table:**

	*x*	*y*	*z*	*U*_iso_*/*U*_eq_	Occ. (<1)
V1	0.3333	0.6667	0.019498 (13)	0.00690 (6)	
Na1	0.3333	0.6667	0.1667	0.149 (5)	0.805 (18)
Na2	0.6667	0.96726 (19)	0.0833	0.0522 (10)	0.731 (7)
P1	−0.04273 (5)	0.3333	0.0833	0.00866 (8)	
O1	0.14193 (13)	0.49765 (14)	0.07762 (5)	0.01643 (16)	
O2	0.54047 (16)	0.84480 (17)	−0.02643 (7)	0.0259 (2)	

### Atomic displacement parameters (Å^2^)

**Table:**

	*U*^11^	*U*^22^	*U*^33^	*U*^12^	*U*^13^	*U*^23^
V1	0.00643 (7)	0.00643 (7)	0.00784 (10)	0.00321 (4)	0	0
Na1	0.218 (8)	0.218 (8)	0.0111 (14)	0.109 (4)	0	0
Na2	0.0224 (8)	0.0170 (5)	0.119 (2)	0.0112 (4)	−0.0354 (11)	−0.0177 (5)
P1	0.00635 (10)	0.00714 (14)	0.01276 (15)	0.00357 (7)	0.00152 (5)	0.00305 (11)
O1	0.0089 (3)	0.0130 (3)	0.0220 (4)	0.0015 (3)	0.0048 (3)	0.0057 (3)
O2	0.0185 (4)	0.0223 (5)	0.0322 (6)	0.0067 (4)	0.0143 (4)	0.0151 (4)

### Geometric parameters (Å, °)

**Table:**

V1—O2^i^	1.9693 (10)	Na2—O1^ii^	2.3883 (12)
V1—O2	1.9693 (11)	Na2—O1^vi^	2.3883 (12)
V1—O2^ii^	1.9693 (10)	Na2—O1^i^	2.4448 (19)
V1—O1^ii^	2.0271 (9)	Na2—O1^vii^	2.4449 (19)
V1—O1	2.0271 (9)	Na2—O2^viii^	2.6280 (16)
V1—O1^i^	2.0271 (9)	Na2—O2	2.6281 (16)
V1—Na2^i^	3.1070 (6)	Na2—O2^ix^	2.8352 (19)
V1—Na2	3.1070 (7)	Na2—O2^x^	2.8352 (19)
V1—Na2^ii^	3.1070 (6)	Na2—P1^xi^	2.9222 (11)
V1—Na1	3.2096 (3)	Na2—P1^ii^	2.9222 (11)
Na1—O1	2.5045 (11)	Na2—P1^i^	2.9968 (17)
Na1—O1^ii^	2.5045 (11)	P1—O2^xii^	1.5227 (12)
Na1—O1^i^	2.5045 (11)	P1—O2^xiii^	1.5227 (12)
Na1—O1^iii^	2.5045 (11)	P1—O1	1.5358 (10)
Na1—O1^iv^	2.5045 (11)	P1—O1^xiv^	1.5359 (10)
Na1—O1^v^	2.5046 (11)	P1—Na2^xv^	2.9222 (11)
Na1—V1^iv^	3.2081 (2)	P1—Na2^i^	2.9222 (11)
Na1—Na2^i^	3.3193 (6)	P1—Na2^ii^	2.9968 (17)
Na1—Na2^v^	3.3193 (6)	O1—Na2^i^	2.3883 (12)
Na1—Na2	3.3193 (6)	O1—Na2^ii^	2.4448 (19)
Na1—Na2^ii^	3.3205 (6)		
			
O2^i^—V1—O2	96.44 (6)	O1^ii^—Na2—O2	68.20 (4)
O2^i^—V1—O2^ii^	96.44 (6)	O1^vi^—Na2—O2	115.98 (4)
O2—V1—O2^ii^	96.44 (6)	O1^i^—Na2—O2	66.40 (4)
O2^i^—V1—O1^ii^	88.22 (5)	O1^vii^—Na2—O2	93.51 (6)
O2—V1—O1^ii^	89.72 (5)	O2^viii^—Na2—O2	157.25 (9)
O2^ii^—V1—O1^ii^	171.80 (6)	O1^ii^—Na2—O2^ix^	54.99 (4)
O2^i^—V1—O1	89.72 (5)	O1^vi^—Na2—O2^ix^	108.39 (6)
O2—V1—O1	171.80 (6)	O1^i^—Na2—O2^ix^	115.94 (4)
O2^ii^—V1—O1	88.22 (5)	O1^vii^—Na2—O2^ix^	151.11 (4)
O1^ii^—V1—O1	85.05 (5)	O2^viii^—Na2—O2^ix^	85.67 (3)
O2^i^—V1—O1^i^	171.80 (6)	O2—Na2—O2^ix^	112.13 (5)
O2—V1—O1^i^	88.22 (5)	O1^ii^—Na2—O2^x^	108.39 (6)
O2^ii^—V1—O1^i^	89.72 (5)	O1^vi^—Na2—O2^x^	54.99 (4)
O1^ii^—V1—O1^i^	85.05 (5)	O1^i^—Na2—O2^x^	151.10 (4)
O1—V1—O1^i^	85.05 (5)	O1^vii^—Na2—O2^x^	115.94 (4)
O1—Na1—O1^ii^	66.33 (3)	O2^viii^—Na2—O2^x^	112.13 (5)
O1—Na1—O1^i^	66.33 (3)	O2—Na2—O2^x^	85.67 (3)
O1^ii^—Na1—O1^i^	66.33 (3)	O2^ix^—Na2—O2^x^	80.45 (7)
O1—Na1—O1^iii^	113.67 (3)	O2^xii^—P1—O2^xiii^	111.67 (12)
O1^ii^—Na1—O1^iii^	180	O2^xii^—P1—O1	106.07 (7)
O1^i^—Na1—O1^iii^	113.67 (3)	O2^xiii^—P1—O1	112.18 (7)
O1—Na1—O1^iv^	180	O2^xii^—P1—O1^xiv^	112.19 (7)
O1^ii^—Na1—O1^iv^	113.67 (3)	O2^xiii^—P1—O1^xiv^	106.07 (7)
O1^i^—Na1—O1^iv^	113.67 (3)	O1—P1—O1^xiv^	108.74 (9)
O1^iii^—Na1—O1^iv^	66.33 (3)	P1—O1—V1	145.95 (7)
O1—Na1—O1^v^	113.67 (3)	P1—O1—Na2^i^	93.73 (6)
O1^ii^—Na1—O1^v^	113.67 (3)	V1—O1—Na2^i^	89.05 (5)
O1^i^—Na1—O1^v^	180	P1—O1—Na2^ii^	94.93 (5)
O1^iii^—Na1—O1^v^	66.33 (3)	V1—O1—Na2^ii^	87.50 (4)
O1^iv^—Na1—O1^v^	66.33 (3)	Na2^i^—O1—Na2^ii^	169.09 (5)
O1^ii^—Na2—O1^vi^	160.52 (9)	P1—O1—Na1	124.53 (6)
O1^ii^—Na2—O1^i^	69.07 (5)	V1—O1—Na1	89.52 (4)
O1^vi^—Na2—O1^i^	130.40 (6)	Na2^i^—O1—Na1	85.40 (5)
O1^ii^—Na2—O1^vii^	130.40 (6)	Na2^ii^—O1—Na1	84.23 (4)
O1^vi^—Na2—O1^vii^	69.07 (5)	P1^xvi^—O2—V1	151.38 (10)
O1^i^—Na2—O1^vii^	61.41 (6)	P1^xvi^—O2—Na2	120.77 (8)
O1^ii^—Na2—O2^viii^	115.98 (4)	V1—O2—Na2	83.72 (5)
O1^vi^—Na2—O2^viii^	68.20 (4)	P1^xvi^—O2—Na2^xvii^	77.85 (5)
O1^i^—Na2—O2^viii^	93.51 (6)	V1—O2—Na2^xvii^	107.30 (6)
O1^vii^—Na2—O2^viii^	66.40 (4)	Na2—O2—Na2^xvii^	113.64 (7)

Symmetry codes: (i) −*y*+1, *x*−*y*+1, *z*; (ii) −*x*+*y*, −*x*+1, *z*; (iii) *x*−*y*+2/3, *x*+1/3, −*z*+1/3; (iv) −*x*+2/3, −*y*+4/3, −*z*+1/3; (v) *y*−1/3, −*x*+*y*+1/3, −*z*+1/3; (vi) *x*−*y*+4/3, −*y*+5/3, −*z*+1/6; (vii) *y*+1/3, *x*+2/3, −*z*+1/6; (viii) −*x*+4/3, −*x*+*y*+2/3, −*z*+1/6; (ix) −*y*+4/3, −*x*+5/3, *z*+1/6; (x) *y*, −*x*+*y*+1, −*z*; (xi) *x*+1, *y*+1, *z*; (xii) *x*−2/3, *x*−*y*+2/3, *z*+1/6; (xiii) *y*−1, −*x*+*y*, −*z*; (xiv) *x*−*y*+1/3, −*y*+2/3, −*z*+1/6; (xv) *x*−1, *y*−1, *z*; (xvi) *x*−*y*+1, *x*+1, −*z*; (xvii) *x*−*y*+1, *x*, −*z*.
